# Friendship in Autism Spectrum Disorder Is Related to Diverse Developmental Changes Between Toddlerhood and Adolescence

**DOI:** 10.1007/s10803-024-06284-8

**Published:** 2024-03-08

**Authors:** Ronit Saban-Bezalel, Esther Ben-Itzchak, Ditza A. Zachor

**Affiliations:** 1 Bruckner Center for Autism Research, Department of Communication Disorders, Ariel University, Ariel, Israel; 2 The Autism Center/ALUT, Department of Pediatrics, Sackler Faculty of Medicine, Shamir (Assaf Harofeh) Medical Center, Tel Aviv University, Zerifin, 70300 Israel

**Keywords:** Autism, Friendship ability, Longitudinal study, Adaptive skills, Social communication, Educational placement, Comorbidities

## Abstract

**Purpose:**

Follow-up studies of children diagnosed with autism spectrum disorder (ASD) in early childhood that focus on friendship formation during adolescence are scarce. The present study focused on exploring characteristics possibly related to the ability to establish friendships during adolescence among children diagnosed with ASD in toddlerhood.

**Methods:**

The cohort included 43 participants who underwent comprehensive assessments during toddlerhood and adolescence. Participants were divided into two groups [Friendship(+)/Friendship(-)] based on (1) adolescent social insight as assessed by professionals and (2) parental and adolescent self-reports regarding having or not having friends. No differences in IQ, ASD symptoms, or adaptive behavior during early childhood were found between the two groups.

**Results:**

Different and better changes in social communication, adaptive socialization, and daily living skills were observed for the Friendship(+) group. Adolescents with ASD in the Friendship(+) group exhibited greater social independence. Attention-deficit/hyperactivity disorder incidence, anxiety symptom severity, and placement in mainstream or special education classes did not differ between the two groups.

**Conclusion:**

This long-term study highlights that for children with ASD, longitudinal growth in social communication and adaptive functioning is possible, highly important for and related to the development of the complex ability to establish friendship.

## Introduction

Autism spectrum disorder (ASD) is characterized by two main symptom clusters: impaired social communication skills and restricted and repetitive behaviors (RRBs) (American Psychiatric Association, 2013). Individuals with ASD are likely to experience persistent decreases in social communication and social interactions across multiple contexts (American Psychiatric Association, 2013), which may affect their ability to engage in intimate and meaningful social interactions (Petrina et al., 2014).

Given the importance of social relationships in development and quality of life and considering the known social difficulties associated with ASD, previous studies have examined the ability of cognitively able youth with ASD to establish social relationships (Bauminger & Kassari, 2000; Petrina et al., 2014). These studies compared children with ASD with neurotypical children and indicated that children with ASD can make friends (Bauminger & Kassari, 2000; Bauminger et al., 2008; Freeman et al., 2015). Similarities in developmental patterns of friendships (Bauminger et al., 2010), friendship expectations (Bottema-Beutel et al., 2019), satisfaction with relationships (Calder et al., 2012), and the desire to have friends (Mendelson et al., 2016) were described among children with ASD and neurotypical children. However, the friendship characteristics of children with ASD differed from those of age-matched neurotypical children in terms of the number of friends, frequency of meetings with friends outside of school, and friendship duration and stability (Mendelson et al., 2016; Petrina et al., 2014). Moreover, even when cognitive levels and verbal skills were controlled for, the quality of friendships of school-age children with ASD was lower than that of their neurotypical peers (Bauminger et al., 2010; Calder et al., 2012; Freeman et al., 2015; Mendelson et al., 2016; Petrina et al., 2014). When asked to define the concept of “friendship,” unlike neurotypical children, children with ASD, provided definitions based more on sharing company and less on emotional connectedness (Calder et al., 2012). Factors identified in late childhood and adolescence that correlated with enhanced friendship abilities in children with ASD included children’s motivations for social contact (Calder et al., 2012), verbal abilities (Jones et al., 2017), ASD symptom severity (Mazurek & Kanne, 2010), and increased participation in social activities (Dovgan & Mazurek, 2019).

Regarding the link between cognitive ability and friendship quality, existing research presents conflicting findings. Mazurek and Kanne (2010) discovered a positive correlation between cognitive ability and friendship quality, indicating that children with higher cognitive abilities tended to have better-quality friendships. Conversely, several other studies, including those by Calder et al. (2012) and Locke et al. (2017), did not find a significant relationship between cognitive ability and friendship quality or social success, concluding that cognitive ability may not be directly related to the capacity for forming or maintaining friendships.

Comorbidities in individuals with ASD can further impair social relationships. ADHD and anxiety, both of which are frequent comorbidities in individuals with ASD, have been reported to be negatively associated with the social domain. Several studies have reported that coexisting ASD and ADHD are associated with greater social difficulties than is ASD alone (Rao & Landa, 2014; Zachor & Ben-Itzchak, 2018; Wang et al., 2023). Several previous studies, although not all, have reported that anxiety in children with ASD has a negative impact on their relationships with peers, teachers, and family members (Bellini, 2006; Factor et al., 2017: McVey et al., 2018). Furthermore, there was evidence of improved social functioning in children with ASD following a cognitive-behavioral intervention that effectively reduced their anxiety (Wood, 2006).

The relationships between external environmental factors, particularly the educational placement of children with ASD (special education vs. inclusive settings), and the development of friendship skills have received limited research attention. One study (Jones et al., 2017) revealed that children with ASD who were integrated into mainstream classrooms exhibited more social connections with their peers than did those in special education classrooms. Conversely, other studies have presented different findings and noted no significant correlation between social outcomes and the type of class attended, whether in specialized ASD or inclusive settings, for both adolescents (Orsmond et al., 2004) and adults (Howlin et al., 2013; Orsmond et al., 2004) with ASD. Choosing whether to enroll children with ASD in specialized ASD classrooms or inclusive settings is a vital decision for parents, given the potential association of the classroom setting with the social outcomes of these children.

Examining the factors influencing friendship outcomes in individuals with ASD is crucial. Early identification of these factors is key to enabling timely, tailored interventions. This study aimed to clarify the existing contradictory findings regarding the relationships among cognitive ability, autism severity, educational placement, and the formation of friendships in adolescents with ASD.

To our knowledge, only two longitudinal follow-up studies have been initiated in early childhood to examine the later social development of individuals with ASD (Friedman et al., 2019; Howlin et al., 2013). One study examined variables related to toddlerhood possibly associated with later friendship quality (Freeman et al., 2015) and found that early (3.6 years of age) core social communication abilities (better joint attention and initial play), but not developmental characteristics (i.e., IQ and receptive and expressive language), were linked to better friendship quality at 8–9 years of age. In their study, Howlin et al. (2013) followed a cohort of 60 children with ASD (mean age of 6.75 years) as they transitioned into adulthood (mean age of 44 years). Despite a documented decrease in autism severity over the intervening years, the study revealed that social outcomes in adulthood were notably less favorable. One intriguing finding was the positive correlation observed between early language ability and social functioning in adulthood. Furthermore, the study noted that the Autism Diagnostic Interview-Revised Reciprocal Social Impairment (ADI–R; Lord et al., 1994) domain score at the time of diagnosis was the most robust predictor of overall outcomes in adulthood.

Given that there has been only one study in the field of ASD investigating the link between characteristics observed in toddlerhood and later friendship outcomes, there is a notable gap in the realm of long-term follow-up studies concerning children with ASD. This gap emphasizes the need for further investigation in this critical area. It is crucial to identify specific traits in early childhood that can be targeted through interventions to promote the development of friendships during adolescence and adulthood. Additionally, understanding the role of comorbidities and external factors, such as the type of educational setting, is essential for tailoring appropriate treatments and creating an educational environment conducive to fostering friendships. To address these significant research gaps, this longitudinal study examined the developmental and behavioral changes observed in children diagnosed with ASD during toddlerhood across time, with a particular emphasis on their ability to form friendships during adolescence.

This study had two main aims:

1. To compare developmental changes in the cognitive ability, ASD symptoms, and adaptive skills of two groups of adolescents diagnosed with ASD during toddlerhood who differed in their ability to establish friendships.
2. To compare the quality of social participation, comorbidities (ADHD and anxiety symptoms), and educational placement between these two groups.

## Methods

### Participants

This study was part of a larger longitudinal research project following an original cohort of 69 adolescents initially diagnosed with ASD as toddlers between 2002 and 2009 at a tertiary “Autism Center” in a large medical center. All parents of these adolescents consented to the use of data gathered from the charts and individual assessments for various research purposes. In the present study, the inclusion criterion for participation required assessment at T2 using the Autism Diagnostic Observation Schedule (ADOS-2; Lord et al., 1999, 2012) modules 3 or 4, which require the ability to form complex sentences. Out of the initial cohort of 69 participants, only 43 met this criterion. All participants were white and of Jewish ethnicity. The participant characteristics are detailed in Table 1.

**Table 1:** Child and maternal demographic characteristics

	Range	Friendship(+) M(SD)	Friendship(-) M(SD)	*F*	*p*	η^2^
ASD diagnostic age (years; *n* = 43) Age at follow-up (years; *n* = 43) T2−T1 (years; *n* = 43)	1.3–3.0 10.5–17.10 8.04–15.06	2.00 (0.3) 13.08 (2.01) 11.08 (2.01)	2.1 (0.4) 13.05 (1.10) 11.04 (1.08)	0.05 0.208 0.56	0.83 0.65 0.62	0.00 0.01 0.01
Maternal age at birth (years; *n* = 42)	25–46	32.08 (5.5)	31.07 (5.0)	0.74	0.40	0.02
Maternal education (years; *n* = 39)	11–22	15.61 (2.77)	14.90 (2.49)	0.71	0.40	0.02

T1- assessment during toddlerhood; T2- follow-up assessment

### Measures

#### Measures Used During Toddlerhood and Adolescence

**Cognitive Functioning.** Cognitive ability during toddlerhood (T1) was assessed using one of the following instruments, depending on the child’s age: the Mullen Scales of Early Learning (MSEL; Mullen, 1995), the Wechsler Preschool and Primary Scale of Intelligence-Revised (WPPSI-R; Wechsler, 1989), the Stanford-Binet Intelligence Scales (Thorndike et al., 1986), or the Bayley Scales of Infant and Toddler Development-2nd Edition (BSID-II; Bayley, 1993). At the follow-up assessment (T2), cognitive evaluation was conducted using either the Wechsler Intelligence Scale for Children-4th Edition (WISC-IV; Wechsler, 2010) or the Wechsler Adult Intelligence Scale (WAIS; Wechsler, 2001), depending on the participant’s age. Depending on the specific test used, either a general developmental quotient (DQ) score or an intelligence quotient (IQ) score was determined.

**Autism Diagnostic Observation Schedule** (ADOS; Lord et al., 1999, 2012). The ADOS is a semi-structured interactive diagnostic assessment designed to assess social and communicative functioning in individuals with ASD. The ADOS total algorithm score is used to calculate the total severity score using the ADOS calibrated severity scale (CSS; Gotham et al., 2009). The scores of each ADOS subdomain, the social affect (SA), and restricted and repetitive behaviors (RRBs) domains are used to calculate symptom severity using the SA-CSS and RRB-CSS scores (Hus et al., 2014); higher scores reflect more severe ASD symptoms.

**Vineland Adaptive Behavior Scales** (VABS; Sparrow et al., 1984, 2005). The VABS involve a standardized caregiver interview designed to assess adaptive behaviors in children from birth to 18 years of age. The scores of the four subdomains (communication, daily living skills, socialization, and motor skills) yield a standard score. A total score is also calculated, namely, the adaptive behavior composite, in which higher scores reflect better functioning. We used the subdomain standard scores in our analysis.

#### Measures Used During Adolescence

**Conners 3rd Edition** (Conners 3; Conners et al., 2011). The latest version of the Conners 3 is used to provide a reliable assessment of ADHD. We used the parents’ and the teachers’ forms. The assessment features multiple content scales that assess ADHD-related concerns such as inattention and hyperactivity/impulsivity (H/I). Symptoms of distinct diagnoses can be assessed with scales that link directly to the DSM-IV-TR (American Psychiatric Association, 2000). The existing criteria for ADHD include receiving a Conners score ≥ 70 from both parents and teachers.

**Screen for Child Anxiety Related Emotional Disorders** (SCARED; Birmaher et al., 1997). This anxiety questionnaire compares multiple perspectives on a child’s levels of anxiety as reported by the child and/or their parent (child-/parent-report versions). In the present study, only the parent-report version was used. This version of the questionnaire has 41 items, which are rated on a scale from 0 to 2 (0 = not true or hardly ever true; 2 = very true or often true). The parent-report version demonstrated excellent internal consistency in the present sample (α (parent-report version) = 0.95).

#### Parent Questionnaire

This questionnaire was developed by two of the authors of this study to solicit information from parents about their adolescents’ medical problems, treatments and educational settings from the time of ASD diagnosis in toddlerhood to adolescence and their current friendships, social participation and leisure time activities. Of this extensive questionnaire, only three parts were used in this study:

1. *Friendship*. (a) The parents were asked whether their child has at least one stable friendship (had no friends = 0, had at least one friend = 1). (b). If parents reported that their child had a friend, they were asked “How many friends does your child have?” (score: number of friends reported by the parents).
2. *Quality of social participation*. The parents were queried about their child’s social participation quality. Using a five-point Likert scale (scores 1–5), the parents expressed the amount of help they believe their child requires to engage in particular social activities, such as attending peers’ birthday parties, class parties, and after-school events. A score of 1 indicated a requirement for complete assistance, while higher scores indicated progressively more independent participation, with a score of 5 indicating no need for assistance.
3. *Educational setting*. Parents were queried about their child’s current or most recent educational setting, which fell into one of two categories: a mainstream educational setting with or without support (score of 1) or a special education setting within a mainstream education setting for children and adolescents with ASD (score of 0).

#### Friendships During Adolescence

To define friendship in this cohort, we used three sources of information to construct the friendship variable. This variable included three components that enabled splitting of the cohort into two groups (Friendship(+)/Friendship(-)): (1) *adolescent self-reports and identification of friendship(s)*: derived from the response of the adolescent when asked to nominate a friend; (2) *parental reports regarding whether their child has a stable friendship*: derived from the Parent Questionnaire (yes/no; scoring 0–1); and (3) *professionals’ assessments of adolescents’ social insight*: determined from ADOS Module 3-Item B6 or Module 4-Item B7 (“Insight into Typical Social Situations and Relationships”), which focus on a participant’s ability to provide spontaneous examples of insight into the nature of various social relationships, including friendships. It is assumed that to form and maintain friendships, children must have adequate social insight. The ADOS score for this item ranges from 0 to 3 based on the participant’s ability to provide examples of such insight; lower scores indicate greater insight into various social relationships. In the present study, for the Friendship variable, a score of 0–1 was considered to indicate adequate social insight, whereas a score of 2–3 indicated insufficient social insight. Note that this ADOS item is not included in the ADOS algorithm and therefore has no impact on the ADOS-CSS score. Of the entire cohort, 39 (90%) participants identified a friend, 32 (74%) parents reported that their child had a friend, and 26 (60%) participants exhibited adequate social relationship insight (with a score of 0–1). Based on the Friendship Variable, the cohort was divided into two groups: a Friendship(+) group, which included participants who reported having a friend, participants whose parents reported that they had at least one steady friendship, and participants who received a score of 0–1 on the ADOS social insight subscale [*n* = 19, female = 2], and a Friendship(-) group, which included participants who did not meet all three criteria [*n* = 24, female = 2].

### Procedure

The parents signed an informed consent form to participate in the study in accordance with the medical center’s Helsinki committee requirements; adolescent participants provided verbal assent. One of the participants, who was older than 18 years, independently signed the consent form. Sessions were held with each participant at two time points: T1, when diagnosed during toddlerhood [mean age 2.1 (0.4) years]; and T2, at follow-up during adolescence [mean age 13.06 (2) years]. At T1, all participants underwent a comprehensive assessment of their behavioral, cognitive, and adaptive skills. Behavioral assessments were conducted by a skilled interdisciplinary team. Pediatric neurologists collected medical, developmental, and familial histories from the parents of the toddlers and conducted a comprehensive neurological examination. The evaluation of ASD was based on two gold standard tests: the Autism Diagnosis Interview-Revised (ADI-R; Lord et al., 1994) and the ADOS (Lord et al., 1999), as well as a clinical judgment based on the DSM-IV criteria (American Psychiatric Association, 2000). Cognitive abilities were assessed using the Mullen Scales of Early Learning (Mullen, 1995), the Wechsler Preschool and Primary Scale of Intelligence-Revised (WPPSI-R; Wechsler, 1989), the Stanford-Binet Intelligence Scales (Thorndike et al., 1986) or the Bayley Scales (Bayley, 1993). Adaptive skills were assessed using the VABS (Sparrow et al., 1984).

At follow-up (T2), each participant met with experienced psychologists who performed a cognitive evaluation using the Wechsler Intelligence Scale for Children-4th Edition (WISC-IV; Wechsler, 2010) or the Wechsler Adult Intelligence Scale (WAIS; Wechsler, 2001) over two visits, according to the participant’s age; ASD symptoms were assessed using the ADOS-2 (Lord et al., 2012). Parents completed the VABS (Sparrow et al., 2005) in a face-to-face interview with a special education expert. Parents completed two questionnaires: the parental version of the Conners 3 (Conners et al., 2011), for the assessment of ADHD symptom severity, and the SCARED (Birmaher et al., 1997), for the assessment of anxiety symptom severity. Teachers completed the teacher’s version of the Conners 3 (Conners et al., 2011) for the assessment of ADHD symptom severity.

### Data Analysis

The normality of the distribution of the variables was examined using skewness and kurtosis tests. Continuous variables related to the participants’ characteristics (IQ score, Vineland Adaptive Behavior Scales (VABS) score; social and daily living skills subdomain scores; ADOS-CSS subdomain scores; social participation; Conners 3 score; and SCARED score) were normally distributed (skewness and kurtosis range = ± 1). Nevertheless, the Vineland Adaptive Behavior Scales (VABS) communication subdomain score exhibited a nonnormal distribution. Therefore, nonparametric analysis was employed for the VABS communication subdomain.

First, we examined the validity of the group classification based on the Friendship variable and other social ability measures through a logistic regression model. The group (Friendship(+)/Friendship(-)) served as the dependent variable, and changes in the ADOS-SA-CSS score over time and changes in the VABS socialization score over time were used as predictors.

We subsequently compared the Friendship(+) and Friendship(-) groups across several demographic characteristics. For T1 and T2, one-way analyses of variance (ANOVAs) were performed for child age at diagnosis and follow-up, years between diagnosis and follow-up, and maternal years of education. We then examined developmental trajectories in ASD symptoms, adaptive behavior (VABS social and daily living skills subdomains), and cognitive ability using a series of 2 × 2 (2 time points × 2 groups) ANOVAs with repeated measures for time. The dependent variables included the developmental quotient (DQ)/IQ scores, each ADOS-CSS subdomain score, the SA and RRB scores (Esler et al., 2015), the VABS socialization score and the VABS daily living skills score. For the VABS communication score, we used a nonparametric Wilcoxon test to measure changes over time in each group separately.

Next, we compared the Friendship(+)/Friendship(-) groups regarding social participation and symptom severity for anxiety (SCARED) using a series of one-way ANOVAs. Nonparametric chi-square analyses were performed for categorical variables, including sex, educational setting (special education classes vs. mainstream education settings), and the presence of ADHD.

## Results

As presented in Table 1, the groups did not significantly differ in terms of participant age at baseline (T1) or follow-up (T2), the time difference between baseline and follow-up, maternal age at birth or maternal education level (see Table 1). There was no significant difference in sex distribution between the groups [χ^2^ (1) = 0.06, *p* = .80].

### The Validity of the Grouping Procedure

Initially, we sought to predict membership in the Friendship group by considering alterations in the ADOS-SA-CSS score and changes in the VABS socialization score from T1 to T2. Our logistic regression model, with membership in the Friendship(+) or Friendship(-) group as the dependent variable and changes in the ADOS-SA-CSS score and VABS socialization score over time as the independent variables, yielded significant results (χ^2^ (2, *N* = 43) = 8, *p* = .02) and explained 27% of the variance (Nagelkerke R^2^). Notably, only the increase in the VABS socialization score over time emerged as a significant predictor of belonging to the Friendship(+) group (*p* = .03, OR = 1.07, 95% CI [1.003, 1.113]).

### Comparisons by Group: ASD Symptoms, Adaptive Skills, and Cognitive Ability

**Fig. 1:**
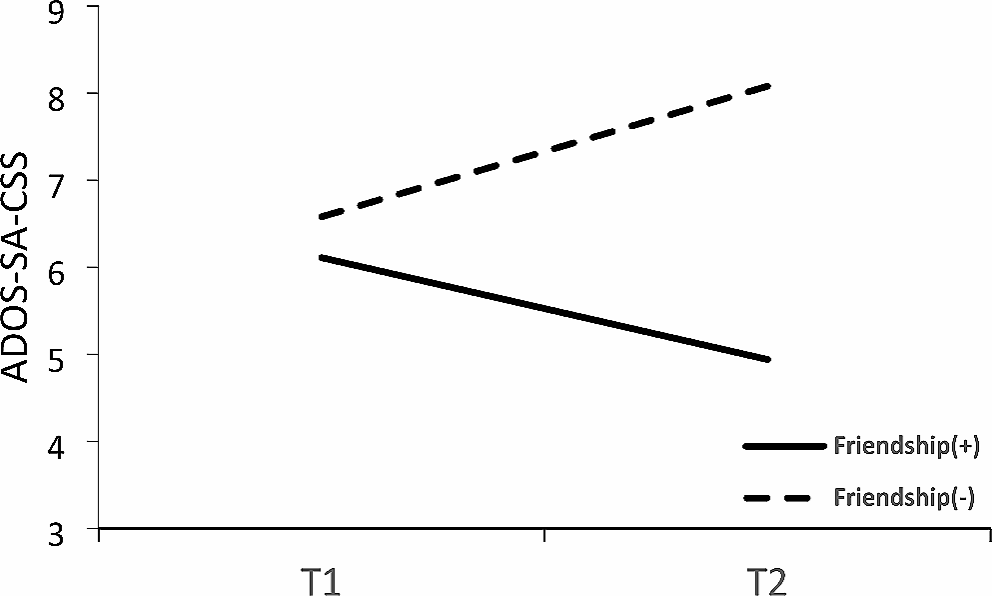
The Autism Diagnostic Observation Schedule (ADOS) social affect (SA) calibrated severity scores (CSS) for both groups at baseline (T1) and follow-up (T2)

We examined the change in the ADOS-SA-CSS score from T1 to T2 for the two defined groups using 2 × 2 ANOVA with repeated measures for time. The analysis yielded a significant main effect for Group with a large effect size (Table 2) but no significant main effect for Time. Figure 1; Table 2 show the severity scores for the Friendship(+) and Friendship(−) groups at T1 and T2. A significant Time × Group interaction with a large effect size (Table 2) was also found. We then separately examined the differences between T1 and T2 for each group. ANOVA revealed a significant increase in the ADOS-SA-CSS score from T1 to T2 in the Friendship(-) group [*F*(1,23) = 4.31, *p* = .049, η^2^ = 0.16], with a large effect size (see Table 2); no significant change in the Friendship(+) group was observed [*F*(1,17) = 1.82, *p* = .19, η^2^ = 0.097]. Hence, while the ADOS-SA-CSS score for the Friendship(-) group increased, there was no significant change in the ADOS-SA-CSS score for the Friendship(+) group over time (Table 2).

**Table 2:** Comparison between the groups regarding the change from T1 to T2 in ASD

	T1 *M(SD)*		T2 *M(SD)*		Time X Group			Time		Group	
	Friendship(+)	Friendship(-)	Friendship(+)	Friendship(-)		**F**	η^2^	**F**	η^2^	**F**	η^2^
ADOS SA-CSS	6.11(2.30)	6.58(2.28)	4.94(3.0)	8.13(2.11)		6.05*	0.13	0.11	0.00	12.82***	0.24
ADOS RRB-CSS	8.00(1.50)	8.75(1.39)	7.22(2.16)	7.87(1.26)		0.024	0.001	7.04*	0.15	3.42	0.08
VABS socialization	71.70(7.53)	69.71(7.87)	93.18(14.29)	80.65(13.98)		5.14*	0.13	48.70***	0.58	6.06*	0.15
VABS daily living skills	69.05(5.71)	67.43(7.25)	91.18(14.82)	79.35(11.59)		4.87*	0.12	54.15***	0.61	7.02*	0.17

ADOS-SA-CSS = Autism Diagnostic Observation Scales – Social Affect – Calibrated Severity Scales; ADOS-RRB-CSS = Autism Diagnostic Observation Scales – Restricted and Repetitive Behavior – Calibrated Severity Scales; VABS Vineland Adaptive Behavior Scale; ADOS-SA-CSS = Autism Diagnostic Observation Scales – Social Affect – Calibrated Severity Scales

For the ADOS-RRB-CSS score, the 2 × 2 ANOVA with repeated measures for time yielded a significant main effect for Time with a large effect size (see Table 2). The significant decrease from T1 to T2 indicated that RRB symptoms improved in both groups at follow-up (T1: M = 8.43, SD = 1.46; T2: M = 7.60, SD = 1.71). No significant Time × Group interaction or main effect for Group was found (Table 2).

**Fig. 2:**
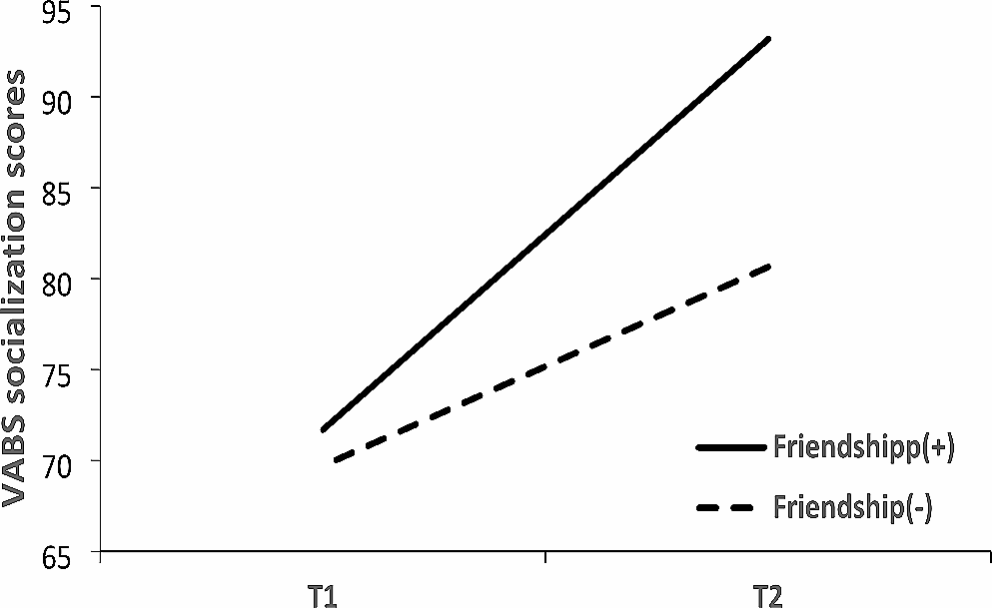
The Vineland Adaptive Behavior Scales (VABS) socialization scores for both groups at baseline (T1) and follow-up (T2)

Next, we examined the change in the VABS socialization score from T1 to T2 for the two defined groups using a 2 × 2 ANOVA with repeated measures for time. The analysis yielded a significant main effect for Group, with a large effect size, and a significant Time effect, with a large effect size. A significant Time × Group interaction with a large effect size was also found (Table 2). Figure 2; Table 2 show the severity scores for the Friendship(+) and Friendship(−) groups at T1 and T2. Examination of the differences between T1 and T2 for each group revealed that social adaptive behavior skills improved in both groups over time. However, independent t tests indicated that the change in the socialization adaptive behavior subdomain score from T1 to T2 was significantly greater in the Friendship(+) group than in the Friendship(-) group [*t*(35) = −2.27, *p* = .03].

**Fig. 3:**
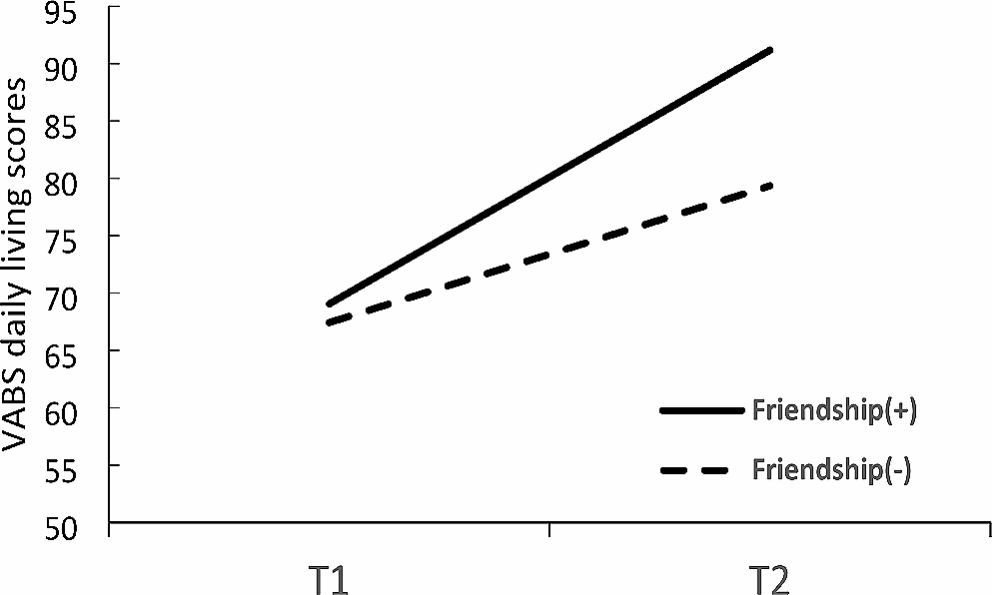
The Vineland Adaptive Behavior Scales (VABS) daily living skills scores for both groups at baseline (T1) and follow-up (T2)

We subsequently examined the change in the VABS daily living skills score from T1 to T2 for the two defined groups using a 2 × 2 ANOVA with repeated measures for time. The analysis yielded a significant main effect for Group, with a large effect size, and a significant Time effect, with a large effect size (Table 2). A significant Time × Group interaction with a large effect size was also found. Figure 3; Table 2 show the severity scores for the Friendship(+) and Friendship(−) groups at T1 and T2. Examination of the differences between T1 and T2 for each group revealed that the daily living behavior skills improved in both groups over time. However, independent t tests indicated that the change in the daily living skills adaptive behavior score from T1 to T2 was significantly greater in the Friendship(+) group than in the Friendship(-) group [*t*(35) = −2.21, *p* = .034]. In summary, while both groups exhibited improvements in social and daily living adaptive skills, the degree of improvement was more pronounced among adolescents with a better ability to establish friendships.

The change in the VABS communication subdomain score between T1 and T2 was examined for each group separately using the Wilcoxon signed rank test. The analysis indicated that scores at T2 (Friendship(+): median = 84, *M* = 87.39, *SD* = 13.03; Friendship(-): median = 89, *M* = 83.70, *SD* = 11.55) were significantly greater than scores at T1 (Friendship(+): median = 70, *M* = 71.94, *SD* = 6.16; Friendship(-): median = 74.5, *M* = 70.64, *SD* = 10.20) for both examined groups (Friendship(+): *Z* = −3.03, *p* = .002; Friendship(-): *Z* = −3.14, *p* = .002).

Finally, we compared the changes in cognitive abilities from T1 to T2 between the two groups. For the DQ/IQ scores, the 2 × 2 ANOVA with repeated measures for time yielded no significant main effect for Time [*F*(1,40) = 0.08, *p* = .78, η^2^ = 0.002] or Group [*F*(1,40) = 1.50, *p* = .23, η^2^ = 0.04] and revealed no significant Time × Group interaction [*F*(1,40) = 3. 11, *p* = .09, η^2^ = 0.08]. There was no significant difference between IQ scores at T1 and T2 (T1: *M* = 77.93, *SD* = 20.30; T2: *M* = 77.74, *SD* = 18.88) for either group.

In summary, the examination of developmental changes from toddlerhood to adolescence within each group revealed noteworthy distinctions between the Friendship(-) and Friendship(+) groups. The Friendship(-) group exhibited more severe social communication symptoms, whereas the Friendship(+) group displayed no significant changes in this domain. However, both groups exhibited similar reductions in symptom severity related to repetitive and restrictive behaviors. Regarding adaptive behavior skills, significant improvements were evident in both groups, particularly in the areas of socialization and daily living skills. Notably, these improvements were more pronounced in the Friendship(+) group. Additionally, both groups demonstrated similar improvements in their communication skills. Finally, in terms of cognitive ability, no significant changes were detected in either group.

For the second aim, we examined differences between the two groups (Friendship(+)/Friendship(-)) at T2 across several domains: social participation, educational setting, and comorbidity (ADHD and anxiety symptom severity).

### Quality of Social Participation

The level of assistance the children needed to participate in specific social activities (i.e., birthday parties, after-school activities) was compared between the two groups. The Friendship(+) group was more socially independent [*F*(1,40) = 5.83, *p* = .02, η^2^ = 0.13] and needed less assistance to participate in social activities (*M* = 3.8, *SD* = 0.72) than did the Friendship(-) group (*M* = 3.14, *SD* = 1.02).

#### Educational Setting at T2

The groups’ educational settings (mainstream settings vs. special education classes) were also examined. No statistically significant difference was found between the groups [χ^2^ (1) = 0.09, *p* = .79], indicating a similar distribution of educational settings for the two groups.

#### ADHD and Anxiety Comorbidities at T2

Finally, we evaluated the two common comorbidities associated with ASD—ADHD and anxiety—in the Friendship(+)/Friendship(-) groups at T2. For ADHD, 32% of the Friendship(+) group and 38% of the Friendship(-) group met the criteria for ADHD based on Conners 3 scores from parents and teachers, with no significant difference between the groups [χ^2^ (1) = 0.16, *p* = .69]. For anxiety symptoms, ANOVA yielded no significant group effect for the SCARED total scores from parents [*F*(1,41) = 1.92, *p* = .17, η^2^ = 0.05].

In summary, the results pertaining to our second aim suggest that concerning educational settings, the two groups did not show significant differences in the frequency of enrollment in mainstream or special education classes. Additionally, no significant disparities were observed between the two friendship groups in terms of comorbidities, including the severity of ADHD or anxiety symptoms. However, a noteworthy distinction emerged in the domain of social participation, particularly regarding the level of assistance needed for engagement in social activities. Adolescents in the Friendship(+) group displayed a greater degree of social independence.

## Discussion

Forming social relationships is a fundamental human activity that positively affects development and quality of life. Children and adolescents with ASD may experience challenges in their perceptions of the social realm and in establishing and maintaining friendships.

The initial objective of this distinctive long-term follow-up study was to examine developmental changes in cognitive ability, ASD symptoms, and adaptive skills among children diagnosed with ASD in toddlerhood, with a focus on their ability to establish friendships in adolescence. Interestingly, there were no disparities in cognitive ability, ASD symptoms, or adaptive behaviors during toddlerhood between individuals with a greater ability to form friendships and those with a lower ability to form friendships. A decade after the initial assessments, there was a general improvement in adaptive communication skills and a reduction in restricted and repetitive behavior symptoms for the entire sample, regardless of their ability to establish friendships during adolescence. Furthermore, the change in cognitive ability did not significantly differ between individuals with a greater ability to form friendships and those with a lower ability to form friendships.

A distinct developmental change was observed for the change in social communication deficits during follow-up, with the group with lower friendship skills displaying more severe symptoms than the group with a greater ability to form friendships. Furthermore, both friendship ability groups demonstrated improvements in adaptive socialization and daily living skills over time, with adolescents with a greater friendship ability experiencing greater gains.

The study’s second aim was to compare the quality of social participation, the presence of comorbidities (ADHD and anxiety symptoms), and educational placement between adolescents with a greater ability to form friendships and those with a lower ability to form friendships. Adolescents with ASD and a greater ability to form friendships were more involved in social participation than were those with a lower ability to form friendships. These adolescents were more socially independent and needed less assistance during social meetings. The present study demonstrated that the formation of friendships and engagement in social activities were not associated with the prevalence or severity of comorbidities, including ADHD and anxiety, or with the type of educational setting, whether mainstream settings or special education classes for children with ASD.

Social communication skills are related to the ability to form social relationships with peers. Therefore, positive changes in social communication abilities among children with ASD are expected to translate into a better friendship ability through the years, as found in this study. Previous studies have linked IQ, language, and communication abilities, along with ASD symptoms at diagnosis, to outcomes in social relationships and the formation of friendships (Friedman et al., 2019; Howlin et al., 2013; Mazurek & Kanne, 2010). Specifically, Mazurek and Kanne (2010) reported that more severe ASD symptoms (as measured by the Autism Diagnostic Interview-Revised (ADI-R) were associated with a lower likelihood of having friends. However, none of these studies examined changes in social impairments in children with ASD in the years after diagnosis.

One especially positive result in this study relates to the gains in adaptive socialization skills for adolescents with ASD who had a greater friendship ability. Although the level of social communication symptoms did not improve significantly from toddlerhood to adolescence in children with ASD, this group acquired significant adaptive socialization skills over time, which may have helped them improve their ability to form friendships. Notably, standard scores for adaptive socialization skills were utilized, suggesting that the increase in these skills for participants with a greater ability to form friendships was above what was expected according to their baseline developmental curve. This finding is encouraging considering previous data indicating that adaptive behavior skills, specifically socialization skills, decrease with age in individuals with ASD (Kanne et al., 2011).

Daily living skills relate to a wide range of behaviors and skills required for self-care and participation in community activities, which are important for the development of independence. However, in most studies on social relationships and friendships among individuals with ASD, these skills were not the research focus, except in one study reporting that daily living skills predict social participation in adolescents and adults (Orsmond et al., 2004). Our findings align with these results, indicating that adolescents with ASD whose daily living skills improved were more independent in terms of social participation (i.e., lower levels of assistance needed to participate in various social activities). Thus, daily living skills may be crucial to the formation of friendships.

Unlike the aforementioned variables, the change in cognitive ability over time was not different between adolescents with higher and lower abilities to form friendships. This finding is consistent with that of Freeman et al. (2015), who indicated that the early development of the social communication skills of joint attention and play was more related to friendship quality than other characteristics, such as IQ and the use of receptive and expressive language. These findings may emphasize the notion that, among children and adolescents with autism, the difficulties in forming friendships stem from innate social difficulties beyond the contribution of the cognition level.

The frequency of ADHD did not differ between individuals with higher or lower abilities to form friendships, despite the existing evidence that the presence of both ADHD and ASD may contribute to social difficulties (Humphreys et al., 2016; Marton et al., 2015; Zachor & Ben-Itzchak, 2018). Similarly, in this study, the severity of anxiety symptoms did not differ across friendship ability levels. However, there is controversy in the literature regarding the relationship between anxiety and social engagement. While some studies have shown that anxiety may contribute to impaired social communication, leading to the avoidance of social situations and thereby reducing opportunities to practice social skills (Duvekot et al., 2018), others (White & Roberson-Nay, 2009; White et al., 2014) have indicated that adolescents with better social insight may be more aware of their social difficulties and differences, leading to higher anxiety levels. These findings may indicate that other factors, likely more closely linked to social communication abilities and independent skills, have a stronger influence on the ability to form friendships than comorbidities.

The study suggested that adolescents with ASD can develop friendships regardless of their educational placement, whether in mainstream settings or special education classes for children with ASD. These findings suggest that each educational setting offers distinct advantages, which could help explain the absence of differences between these settings. For example, autism-specific classes offer valuable support tailored to the specific needs, abilities, and learning styles of children with autism, aiding in the development of competent interactions and potentially fostering friendships. However, mainstream placement provides increased opportunities for social interactions with peers and engagement in community-based social activities.

In summary, this study highlights the possible ability of certain adolescents with ASD to develop friendships and classified participants into groups characterized by higher and lower abilities to form friendships during adolescence. This research unveiled distinct, divergent developmental changes, particularly concerning social communication impairments and adaptive skills in socialization and daily living, which set these two groups apart. Individuals with better friendship outcomes demonstrated greater improvements in these adaptive skill areas, less severe social-communication impairments, and more active social participation. Comorbidities such as ADHD and anxiety, as well as the type of educational setting, showed no significant association with friendship outcomes.

### Limitations and Future Directions

This study identified several factors related to friendship outcomes. However, it is important to note that the data are based on a comparison of two groups, and caution should be exercised when making causal inferences. We acknowledge that our study cohort, while reflecting the naturally occurring sex ratio of male to female individuals with ASD, comprised a high proportion of males, rendering comparisons between sexes regarding friendship ability unfeasible. Therefore, caution should be exercised when attempting to extrapolate our findings to females with ASD.

This study revealed that specific developmental changes that may be associated with the ability to form friendships in adolescence. To enhance the generalizability of our findings to the broader autism community, we recommend that future follow-up studies replicate the study protocol across diverse ethnic populations. Furthermore, incorporating additional evaluation measures of comorbidities, including direct examinations, could provide a more in-depth understanding of the factors influencing friendship formation by individuals with ASD. Additionally, future studies should explore developmental language changes over the years, a dimension that was not evaluated in the present study but is potentially linked to friendship outcomes. Investigating the predictors and current factors that directly influence the quality of friendships is of significant interest and can contribute to the development of targeted interventions. Additionally, assessing the long-term relationship between the effects of early interventions and treatments on friendship outcomes, which has not been explored thus far, holds substantial importance.

This study has several important theoretical and clinical implications. Theoretically, these findings suggest that developmental changes in social communication and daily living skills are important factors associated with friendship outcomes. In contrast, developmental changes related to cognitive growth and the regulation of attention and anxiety, as well as those related to restricted and repetitive behaviors, are less strongly associated with socialization outcomes.

The findings of this study have several clinical implications for families and therapists working with individuals with ASD. First, clinicians should recognize that even when the clinical presentation in early childhood varies in severity, improvements in socialization skills over time can lead to the establishment and maintenance of peer relationships and increased engagement in social activities. Our results underscore the importance of enhancing daily living skills over the years. It is plausible that greater independence and the improvement of self-help skills during adolescence facilitate the initiation of social interactions and participation in community-based social activities. Therefore, interventions focused on teaching and practicing self-help skills and social skills may contribute to more favorable friendship outcomes.

Given the increased prevalence of ADHD and anxiety symptoms in individuals with ASD, the finding that these conditions may not impede the ability to form friendships holds substantial value. Finally, based on our findings, children with ASD can cultivate positive relationships with their peers in both special education and mainstream educational settings. As some parents are concerned that specific educational placements might hinder their children’s social relationships with peers, these significant findings may alleviate such apprehensions. The study suggested that enrollment in autism-specific classes may not be related to the ability to form social relationships.

With social participation and friendship being crucial for well-being, it is imperative to continue investigating the factors that may improve social outcomes among children, adolescents, and adults with ASD.
